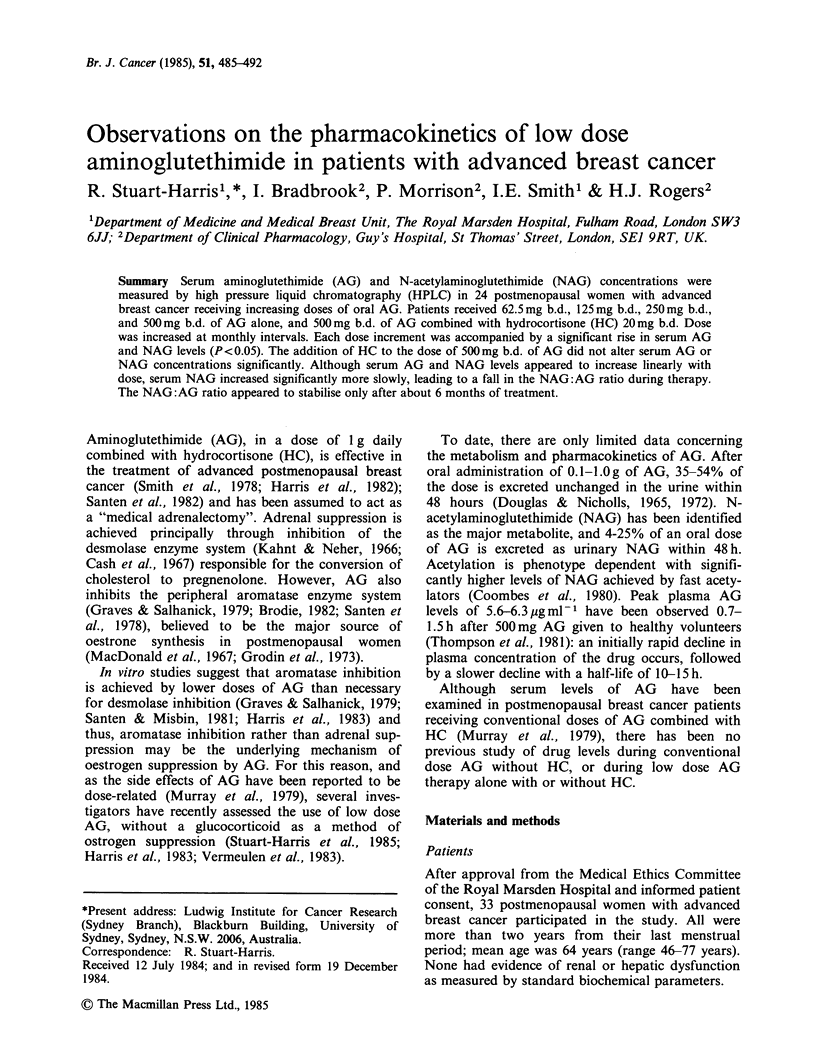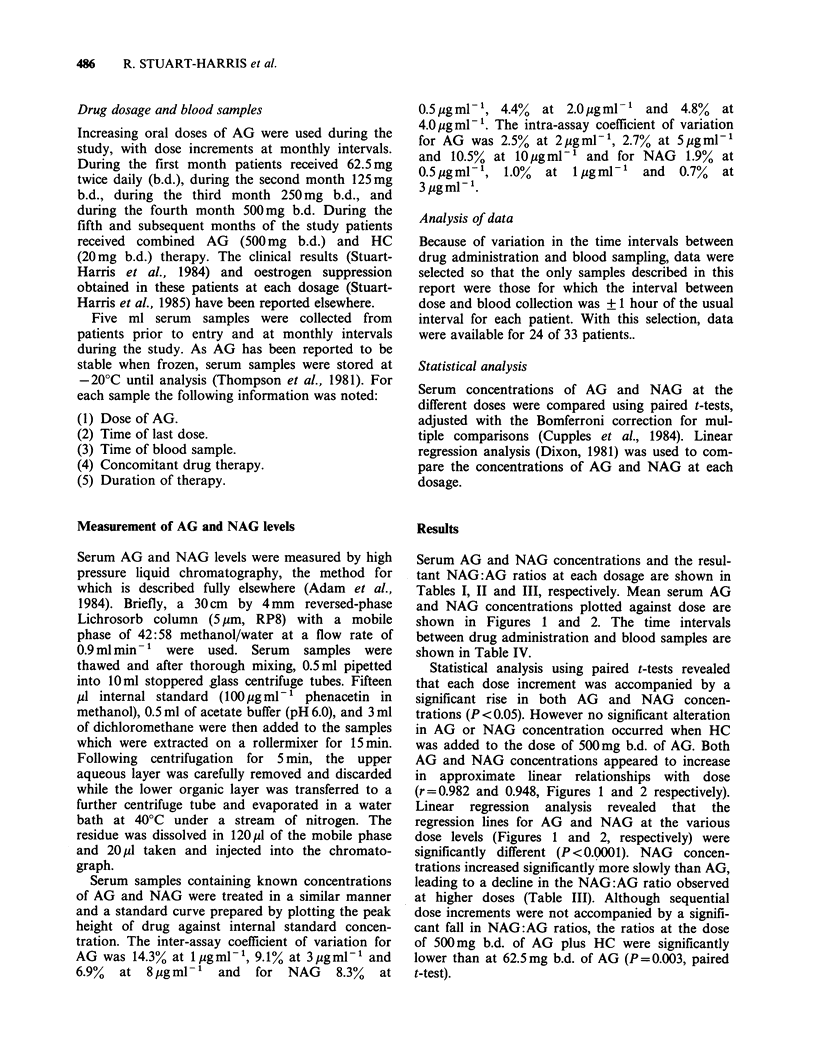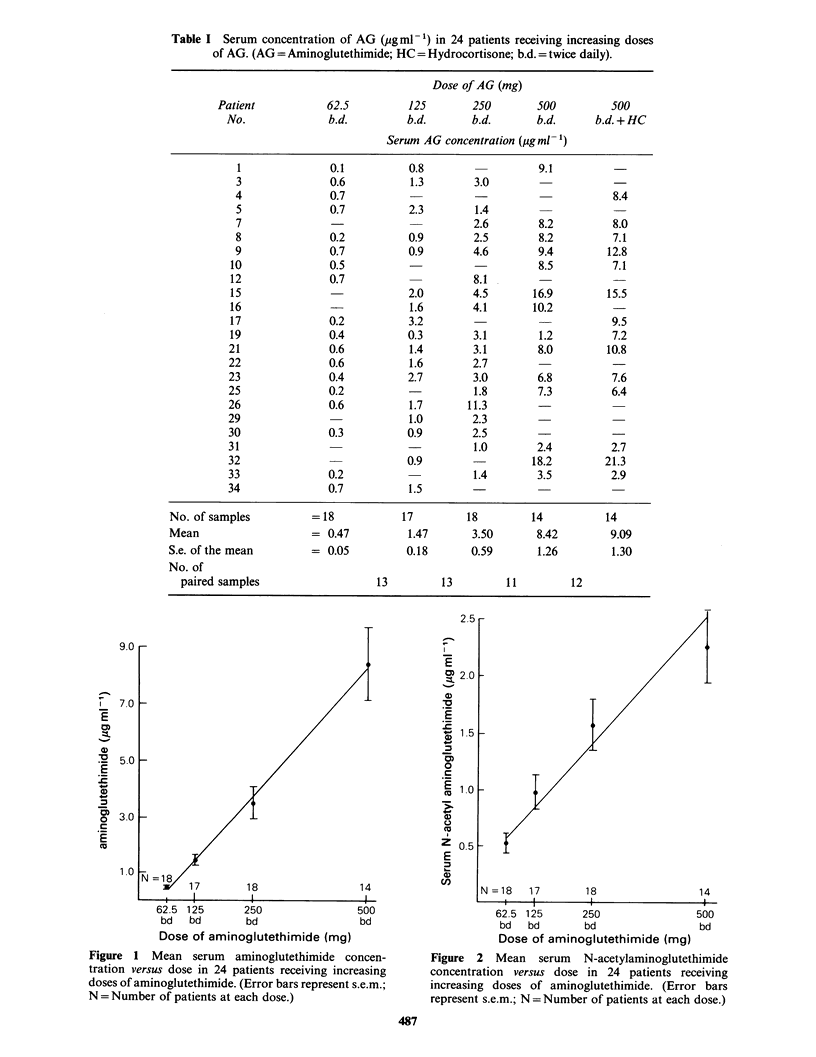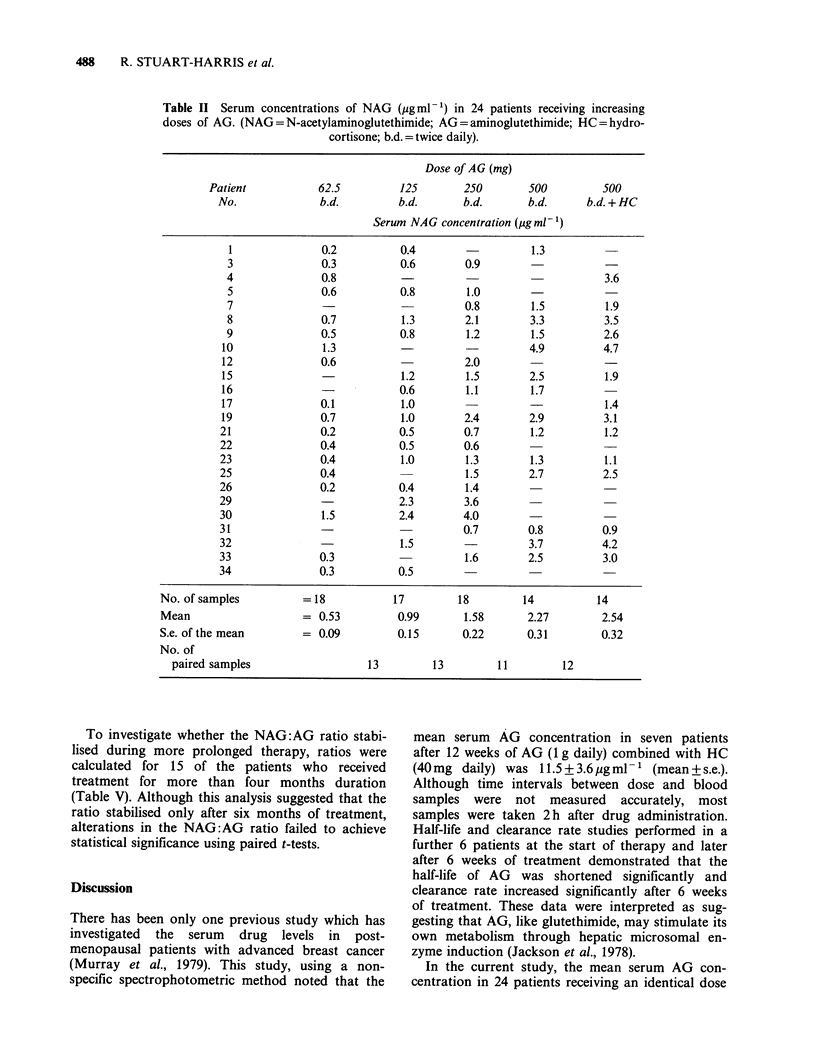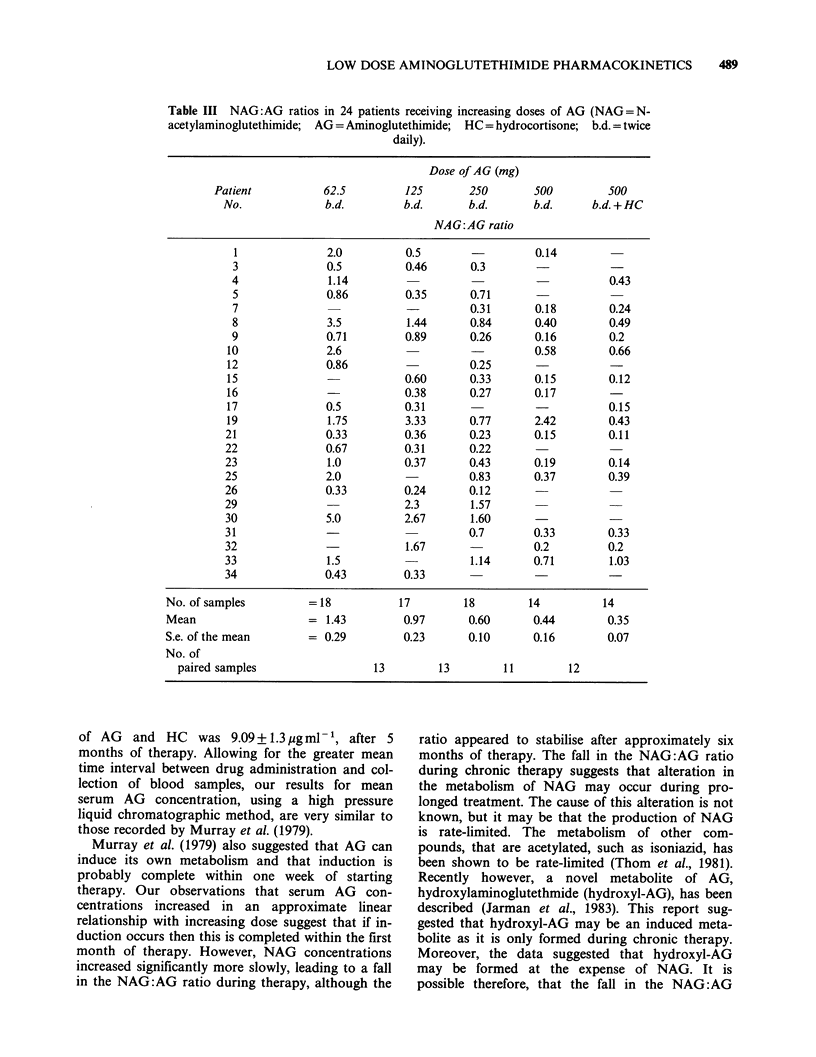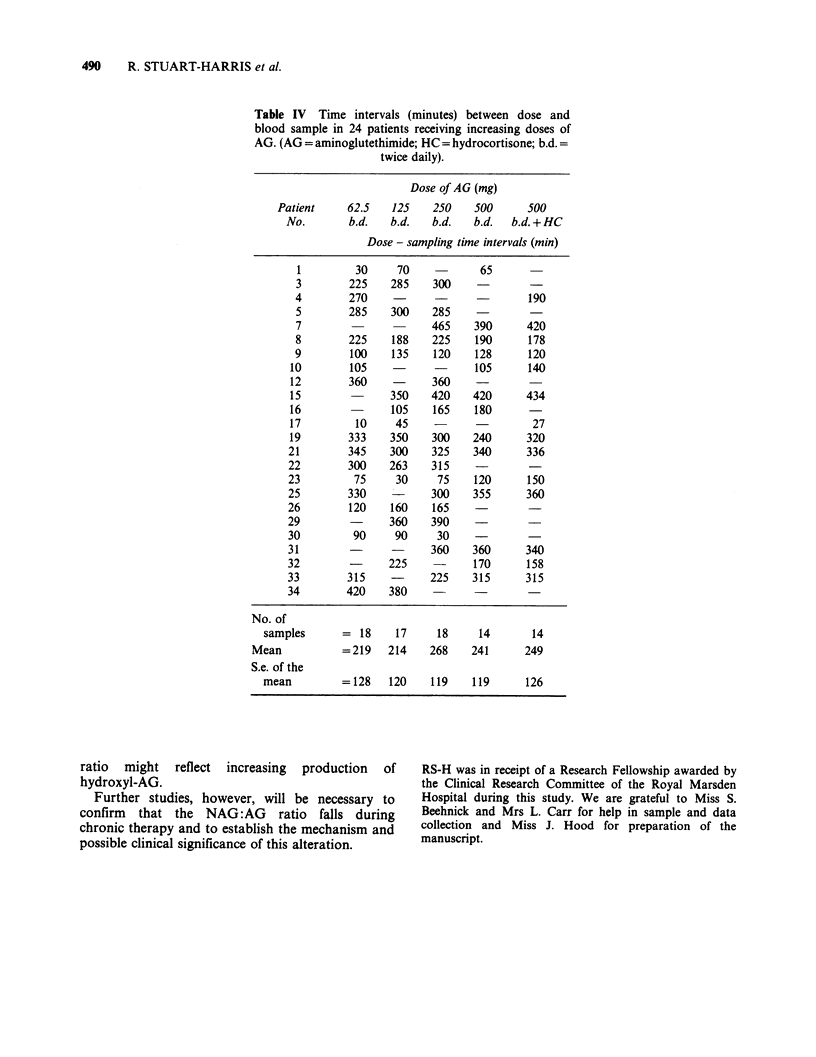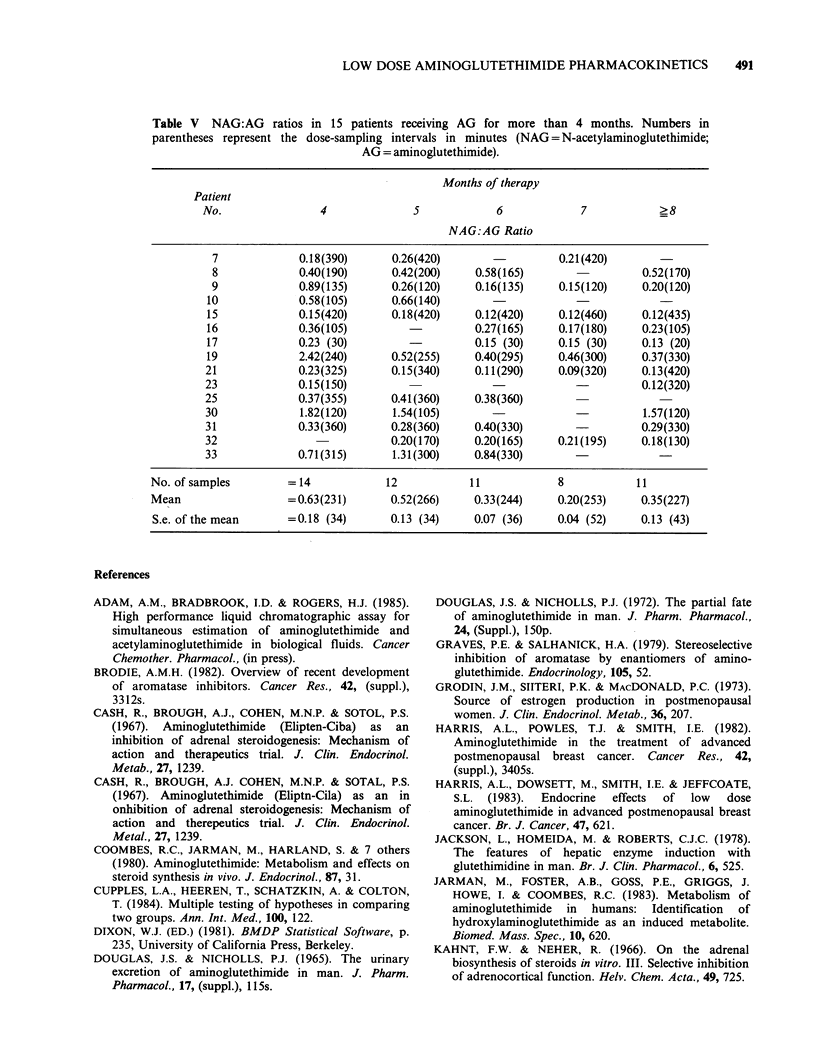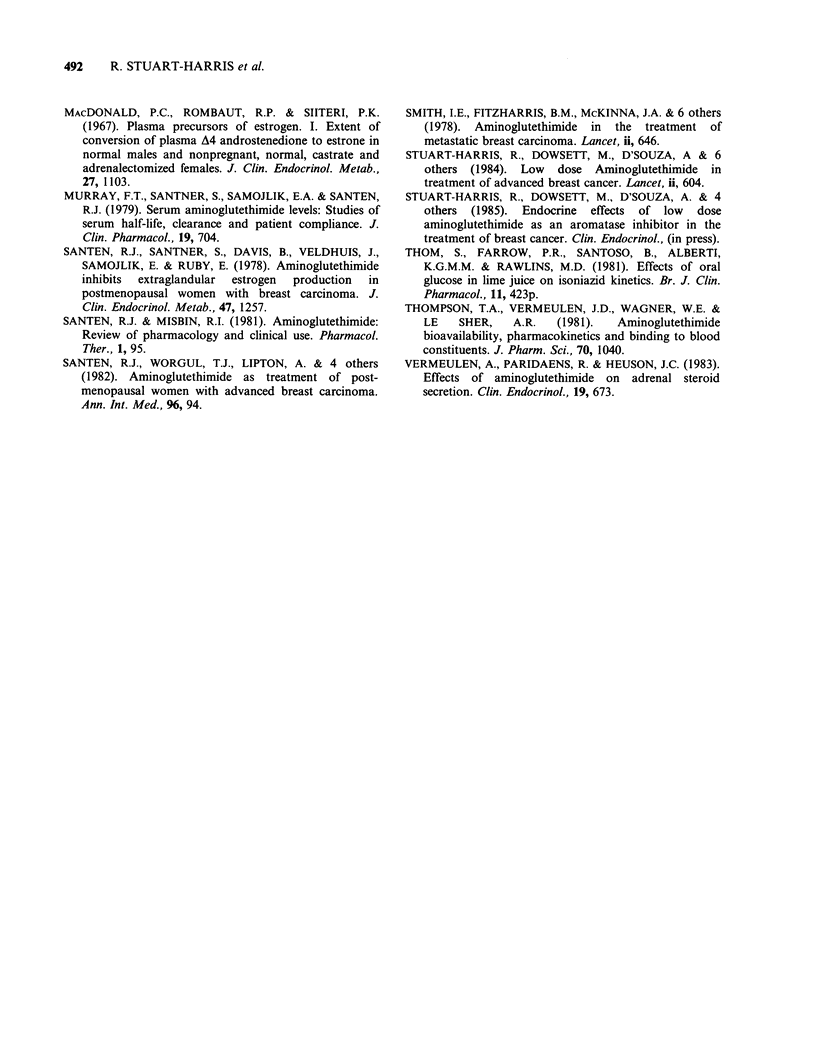# Observations on the pharmacokinetics of low dose aminoglutethimide in patients with advanced breast cancer.

**DOI:** 10.1038/bjc.1985.70

**Published:** 1985-04

**Authors:** R. Stuart-Harris, I. Bradbrook, P. Morrison, I. E. Smith, H. J. Rogers

## Abstract

Serum aminoglutethimide (AG) and N-acetylaminoglutethimide (NAG) concentrations were measured by high pressure liquid chromatography (HPLC) in 24 postmenopausal women with advanced breast cancer receiving increasing doses of oral AG. Patients received 62.5 mg b.d., 125 mg b.d., 250 mg b.d., and 500 mg b.d. of AG alone, and 500 mg b.d. of AG combined with hydrocortisone (HC) 20 mg b.d. Dose was increased at monthly intervals. Each dose increment was accompanied by a significant rise in serum AG and NAG levels (P less than 0.05). The addition of HC to the dose of 500 mg b.d. of AG did not alter serum AG or NAG concentrations significantly. Although serum AG and NAG levels appeared to increase linearly with dose, serum NAG increased significantly more slowly, leading to a fall in the NAG:AG ratio during therapy. The NAG:AG ratio appeared to stabilise only after about 6 months of treatment.


Br. J. Cancer (1985), 51, 485–492

### Observations on the pharmacokinetics of low dose aminoglutethimide in patients with advanced breast cancer

R. Stuart-Harris[1],*, I. Bradbrook[2], P. Morrison[2], I.E. Smith[1] & H.J. Rogers[2]

[1]*Department of Medicine and Medical Breast Unit, The Royal Marsden Hospital, Fulham Road, London SW3 6JJ;* [2]*Department of Clinical Pharmacology, Guy's Hospital, St Thomas' Street, London, SE1 9RT, UK.*

**Summary** Serum aminoglutethimide (AG) and N-acetylaminoglutethimide (NAG) concentrations were measured by high pressure liquid chromatography (HPLC) in 24 postmenopausal women with advanced breast cancer receiving increasing doses of oral AG. Patients received 62.5 mg b.d., 125 mg b.d., 250 mg b.d., and 500 mg b.d. of AG alone, and 500 mg b.d. of AG combined with hydrocortisone (HC) 20 mg b.d. Dose was increased at monthly intervals. Each dose increment was accompanied by a significant rise in serum AG and NAG levels ($P<0.05$). The addition of HC to the dose of 500 mg b.d. of AG did not alter serum AG or NAG concentrations significantly. Although serum AG and NAG levels appeared to increase linearly with dose, serum NAG increased significantly more slowly, leading to a fall in the NAG:AG ratio during therapy. The NAG:AG ratio appeared to stabilise only after about 6 months of treatment.

Aminoglutethimide (AG), in a dose of 1 g daily combined with hydrocortisone (HC), is effective in the treatment of advanced postmenopausal breast cancer (Smith *et al.*, 1978; Harris *et al.*, 1982); Santen *et al.*, 1982) and has been assumed to act as a "medical adrenalectomy". Adrenal suppression is achieved principally through inhibition of the desmolase enzyme system (Kahnt & Neher, 1966; Cash *et al.*, 1967) responsible for the conversion of cholesterol to pregnenolone. However, AG also inhibits the peripheral aromatase enzyme system (Graves & Salhanick, 1979; Brodie, 1982; Santen *et al.*, 1978), believed to be the major source of oestrone synthesis in postmenopausal women (MacDonald *et al.*, 1967; Grodin *et al.*, 1973).

*In vitro* studies suggest that aromatase inhibition is achieved by lower doses of AG than necessary for desmolase inhibition (Graves & Salhanick, 1979; Santen & Misbin, 1981; Harris *et al.*, 1983) and thus, aromatase inhibition rather than adrenal suppression may be the underlying mechanism of oestrogen suppression by AG. For this reason, and as the side effects of AG have been reported to be dose-related (Murray *et al.*, 1979), several investigators have recently assessed the use of low dose AG, without a glucocorticoid as a method of ostrogen suppression (Stuart-Harris *et al.*, 1985; Harris *et al.*, 1983; Vermeulen *et al.*, 1983).

*Present address: Ludwig Institute for Cancer Research (Sydney Branch), Blackburn Building, University of Sydney, Sydney, N.S.W. 2006, Australia.
Correspondence: R. Stuart-Harris.
Received 12 July 1984; and in revised form 19 December 1984.

© The Macmillan Press Ltd., 1985

To date, there are only limited data concerning the metabolism and pharmacokinetics of AG. After oral administration of 0.1–1.0 g of AG, 35–54% of the dose is excreted unchanged in the urine within 48 hours (Douglas & Nicholls, 1965, 1972). N-acetylaminoglutethimide (NAG) has been identified as the major metabolite, and 4-25% of an oral dose of AG is excreted as urinary NAG within 48 h. Acetylation is phenotype dependent with significantly higher levels of NAG achieved by fast acetylators (Coombes *et al.*, 1980). Peak plasma AG levels of 5.6–6.3 $\mu g\,ml^{-1}$ have been observed 0.7–1.5 h after 500 mg AG given to healthy volunteers (Thompson *et al.*, 1981): an initially rapid decline in plasma concentration of the drug occurs, followed by a slower decline with a half-life of 10–15 h.

Although serum levels of AG have been examined in postmenopausal breast cancer patients receiving conventional doses of AG combined with HC (Murray *et al.*, 1979), there has been no previous study of drug levels during conventional dose AG without HC, or during low dose AG therapy alone with or without HC.

### Materials and methods

### *Patients*

After approval from the Medical Ethics Committee of the Royal Marsden Hospital and informed patient consent, 33 postmenopausal women with advanced breast cancer participated in the study. All were more than two years from their last menstrual period; mean age was 64 years (range 46–77 years). None had evidence of renal or hepatic dysfunction as measured by standard biochemical parameters.

*Drug dosage and blood samples*

Increasing oral doses of AG were used during the study, with dose increments at monthly intervals. During the first month patients received 62.5 mg twice daily (b.d.), during the second month 125 mg b.d., during the third month 250 mg b.d., and during the fourth month 500 mg b.d. During the fifth and subsequent months of the study patients received combined AG (500 mg b.d.) and HC (20 mg b.d.) therapy. The clinical results (Stuart-Harris *et al.*, 1984) and oestrogen suppression obtained in these patients at each dosage (Stuart-Harris *et al.*, 1985) have been reported elsewhere.

Five ml serum samples were collected from patients prior to entry and at monthly intervals during the study. As AG has been reported to be stable when frozen, serum samples were stored at $-20°C$ until analysis (Thompson *et al.*, 1981). For each sample the following information was noted:

(1) Dose of AG.
(2) Time of last dose.
(3) Time of blood sample.
(4) Concomitant drug therapy.
(5) Duration of therapy.

**Measurement of AG and NAG levels**

Serum AG and NAG levels were measured by high pressure liquid chromatography, the method for which is described fully elsewhere (Adam *et al.*, 1984). Briefly, a 30 cm by 4 mm reversed-phase Lichrosorb column (5 $\mu$m, RP8) with a mobile phase of 42:58 methanol/water at a flow rate of 0.9 ml $min^{-1}$ were used. Serum samples were thawed and after thorough mixing, 0.5 ml pipetted into 10 ml stoppered glass centrifuge tubes. Fifteen $\mu$l internal standard (100 $\mu g\ ml^{-1}$ phenacetin in methanol), 0.5 ml of acetate buffer (pH 6.0), and 3 ml of dichloromethane were then added to the samples which were extracted on a rollermixer for 15 min. Following centrifugation for 5 min, the upper aqueous layer was carefully removed and discarded while the lower organic layer was transferred to a further centrifuge tube and evaporated in a water bath at 40°C under a stream of nitrogen. The residue was dissolved in 120 $\mu$l of the mobile phase and 20 $\mu$l taken and injected into the chromatograph.

Serum samples containing known concentrations of AG and NAG were treated in a similar manner and a standard curve prepared by plotting the peak height of drug against internal standard concentration. The inter-assay coefficient of variation for AG was 14.3% at 1 $\mu g\ ml^{-1}$, 9.1% at 3 $\mu g\ ml^{-1}$ and 6.9% at 8 $\mu g\ ml^{-1}$ and for NAG 8.3% at 0.5 $\mu g\ ml^{-1}$, 4.4% at 2.0 $\mu g\ ml^{-1}$ and 4.8% at 4.0 $\mu g\ ml^{-1}$. The intra-assay coefficient of variation for AG was 2.5% at 2 $\mu g\ ml^{-1}$, 2.7% at 5 $\mu g\ ml^{-1}$ and 10.5% at 10 $\mu g\ ml^{-1}$ and for NAG 1.9% at 0.5 $\mu g\ ml^{-1}$, 1.0% at 1 $\mu g\ ml^{-1}$ and 0.7% at 3 $\mu g\ ml^{-1}$.

*Analysis of data*

Because of variation in the time intervals between drug administration and blood sampling, data were selected so that the only samples described in this report were those for which the interval between dose and blood collection was $\pm 1$ hour of the usual interval for each patient. With this selection, data were available for 24 of 33 patients..

*Statistical analysis*

Serum concentrations of AG and NAG at the different doses were compared using paired *t*-tests, adjusted with the Bomferroni correction for multiple comparisons (Cupples *et al.*, 1984). Linear regression analysis (Dixon, 1981) was used to compare the concentrations of AG and NAG at each dosage.

**Results**

Serum AG and NAG concentrations and the resultant NAG:AG ratios at each dosage are shown in Tables I, II and III, respectively. Mean serum AG and NAG concentrations plotted against dose are shown in Figures 1 and 2. The time intervals between drug administration and blood samples are shown in Table IV.

Statistical analysis using paired *t*-tests revealed that each dose increment was accompanied by a significant rise in both AG and NAG concentrations ($P < 0.05$). However no significant alteration in AG or NAG concentration occurred when HC was added to the dose of 500 mg b.d. of AG. Both AG and NAG concentrations appeared to increase in approximate linear relationships with dose ($r = 0.982$ and 0.948, Figures 1 and 2 respectively). Linear regression analysis revealed that the regression lines for AG and NAG at the various dose levels (Figures 1 and 2, respectively) were significantly different ($P < 0.0001$). NAG concentrations increased significantly more slowly than AG, leading to a decline in the NAG:AG ratio observed at higher doses (Table III). Although sequential dose increments were not accompanied by a significant fall in NAG:AG ratios, the ratios at the dose of 500 mg b.d. of AG plus HC were significantly lower than at 62.5 mg b.d. of AG ($P = 0.003$, paired *t*-test).

**Table I** Serum concentration of AG ($\mu g\,ml^{-1}$) in 24 patients receiving increasing doses of AG. (AG = Aminoglutethimide; HC = Hydrocortisone; b.d. = twice daily).

| | *Dose of AG (mg)* | | | | |
|---|---|---|---|---|---|
| *Patient No.* | *62.5 b.d.* | *125 b.d.* | *250 b.d.* | *500 b.d.* | *500 b.d. + HC* |
| | *Serum AG concentration ($\mu g\,ml^{-1}$)* | | | | |
| 1 | 0.1 | 0.8 | — | 9.1 | — |
| 3 | 0.6 | 1.3 | 3.0 | — | — |
| 4 | 0.7 | — | — | — | 8.4 |
| 5 | 0.7 | 2.3 | 1.4 | — | — |
| 7 | — | — | 2.6 | 8.2 | 8.0 |
| 8 | 0.2 | 0.9 | 2.5 | 8.2 | 7.1 |
| 9 | 0.7 | 0.9 | 4.6 | 9.4 | 12.8 |
| 10 | 0.5 | — | — | 8.5 | 7.1 |
| 12 | 0.7 | — | 8.1 | — | — |
| 15 | — | 2.0 | 4.5 | 16.9 | 15.5 |
| 16 | — | 1.6 | 4.1 | 10.2 | — |
| 17 | 0.2 | 3.2 | — | — | 9.5 |
| 19 | 0.4 | 0.3 | 3.1 | 1.2 | 7.2 |
| 21 | 0.6 | 1.4 | 3.1 | 8.0 | 10.8 |
| 22 | 0.6 | 1.6 | 2.7 | — | — |
| 23 | 0.4 | 2.7 | 3.0 | 6.8 | 7.6 |
| 25 | 0.2 | — | 1.8 | 7.3 | 6.4 |
| 26 | 0.6 | 1.7 | 11.3 | — | — |
| 29 | — | 1.0 | 2.3 | — | — |
| 30 | 0.3 | 0.9 | 2.5 | — | — |
| 31 | — | — | 1.0 | 2.4 | 2.7 |
| 32 | — | 0.9 | — | 18.2 | 21.3 |
| 33 | 0.2 | — | 1.4 | 3.5 | 2.9 |
| 34 | 0.7 | 1.5 | — | — | — |
| No. of samples | = 18 | 17 | 18 | 14 | 14 |
| Mean | = 0.47 | 1.47 | 3.50 | 8.42 | 9.09 |
| S.e. of the mean | = 0.05 | 0.18 | 0.59 | 1.26 | 1.30 |
| No. of paired samples | | 13 | 13 | 11 | 12 |

**Figure 1** Mean serum aminoglutethimide concentration *versus* dose in 24 patients receiving increasing doses of aminoglutethimide. (Error bars represent s.e.m.; N = Number of patients at each dose.)

**Figure 2** Mean serum N-acetylaminoglutethimide concentration *versus* dose in 24 patients receiving increasing doses of aminoglutethimide. (Error bars represent s.e.m.; N = Number of patients at each dose.)

**Table II** Serum concentrations of NAG ($\mu g\,ml^{-1}$) in 24 patients receiving increasing doses of AG. (NAG = N-acetylaminoglutethimide; AG = aminoglutethimide; HC = hydrocortisone; b.d. = twice daily).

| Patient No. | *Dose of AG (mg)* 62.5 b.d. | 125 b.d. | 250 b.d. | 500 b.d. | 500 b.d. + HC |
|---|---|---|---|---|---|
| | *Serum NAG concentration ($\mu g\,ml^{-1}$)* | | | | |
| 1 | 0.2 | 0.4 | — | 1.3 | — |
| 3 | 0.3 | 0.6 | 0.9 | — | — |
| 4 | 0.8 | — | — | — | 3.6 |
| 5 | 0.6 | 0.8 | 1.0 | — | — |
| 7 | — | — | 0.8 | 1.5 | 1.9 |
| 8 | 0.7 | 1.3 | 2.1 | 3.3 | 3.5 |
| 9 | 0.5 | 0.8 | 1.2 | 1.5 | 2.6 |
| 10 | 1.3 | — | — | 4.9 | 4.7 |
| 12 | 0.6 | — | 2.0 | — | — |
| 15 | — | 1.2 | 1.5 | 2.5 | 1.9 |
| 16 | — | 0.6 | 1.1 | 1.7 | — |
| 17 | 0.1 | 1.0 | — | — | 1.4 |
| 19 | 0.7 | 1.0 | 2.4 | 2.9 | 3.1 |
| 21 | 0.2 | 0.5 | 0.7 | 1.2 | 1.2 |
| 22 | 0.4 | 0.5 | 0.6 | — | — |
| 23 | 0.4 | 1.0 | 1.3 | 1.3 | 1.1 |
| 25 | 0.4 | — | 1.5 | 2.7 | 2.5 |
| 26 | 0.2 | 0.4 | 1.4 | — | — |
| 29 | — | 2.3 | 3.6 | — | — |
| 30 | 1.5 | 2.4 | 4.0 | — | — |
| 31 | — | — | 0.7 | 0.8 | 0.9 |
| 32 | — | 1.5 | — | 3.7 | 4.2 |
| 33 | 0.3 | — | 1.6 | 2.5 | 3.0 |
| 34 | 0.3 | 0.5 | — | — | — |
| No. of samples | = 18 | 17 | 18 | 14 | 14 |
| Mean | = 0.53 | 0.99 | 1.58 | 2.27 | 2.54 |
| S.e. of the mean | = 0.09 | 0.15 | 0.22 | 0.31 | 0.32 |
| No. of paired samples | | 13 | 13 | 11 | 12 |

To investigate whether the NAG:AG ratio stabilised during more prolonged therapy, ratios were calculated for 15 of the patients who received treatment for more than four months duration (Table V). Although this analysis suggested that the ratio stabilised only after six months of treatment, alterations in the NAG:AG ratio failed to achieve statistical significance using paired *t*-tests.

### Discussion

There has been only one previous study which has investigated the serum drug levels in post-menopausal patients with advanced breast cancer (Murray *et al.*, 1979). This study, using a non-specific spectrophotometric method noted that the mean serum AG concentration in seven patients after 12 weeks of AG (1 g daily) combined with HC (40 mg daily) was $11.5 \pm 3.6\,\mu g\,ml^{-1}$ (mean ± s.e.). Although time intervals between dose and blood samples were not measured accurately, most samples were taken 2 h after drug administration. Half-life and clearance rate studies performed in a further 6 patients at the start of therapy and later after 6 weeks of treatment demonstrated that the half-life of AG was shortened significantly and clearance rate increased significantly after 6 weeks of treatment. These data were interpreted as suggesting that AG, like glutethimide, may stimulate its own metabolism through hepatic microsomal enzyme induction (Jackson *et al.*, 1978).

In the current study, the mean serum AG concentration in 24 patients receiving an identical dose

**Table III** NAG:AG ratios in 24 patients receiving increasing doses of AG (NAG = N-acetylaminoglutethimide; AG = Aminoglutethimide; HC = hydrocortisone; b.d. = twice daily).

| *Patient No.* | *62.5 b.d.* | *125 b.d.* | *250 b.d.* | *500 b.d.* | *500 b.d. + HC* |
|---|---|---|---|---|---|
| | *Dose of AG (mg)* | | | | |
| | *NAG:AG ratio* | | | | |
| 1 | 2.0 | 0.5 | — | 0.14 | — |
| 3 | 0.5 | 0.46 | 0.3 | — | — |
| 4 | 1.14 | — | — | — | 0.43 |
| 5 | 0.86 | 0.35 | 0.71 | — | — |
| 7 | — | — | 0.31 | 0.18 | 0.24 |
| 8 | 3.5 | 1.44 | 0.84 | 0.40 | 0.49 |
| 9 | 0.71 | 0.89 | 0.26 | 0.16 | 0.2 |
| 10 | 2.6 | — | — | 0.58 | 0.66 |
| 12 | 0.86 | — | 0.25 | — | — |
| 15 | — | 0.60 | 0.33 | 0.15 | 0.12 |
| 16 | — | 0.38 | 0.27 | 0.17 | — |
| 17 | 0.5 | 0.31 | — | — | 0.15 |
| 19 | 1.75 | 3.33 | 0.77 | 2.42 | 0.43 |
| 21 | 0.33 | 0.36 | 0.23 | 0.15 | 0.11 |
| 22 | 0.67 | 0.31 | 0.22 | — | — |
| 23 | 1.0 | 0.37 | 0.43 | 0.19 | 0.14 |
| 25 | 2.0 | — | 0.83 | 0.37 | 0.39 |
| 26 | 0.33 | 0.24 | 0.12 | — | — |
| 29 | — | 2.3 | 1.57 | — | — |
| 30 | 5.0 | 2.67 | 1.60 | — | — |
| 31 | — | — | 0.7 | 0.33 | 0.33 |
| 32 | — | 1.67 | — | 0.2 | 0.2 |
| 33 | 1.5 | — | 1.14 | 0.71 | 1.03 |
| 34 | 0.43 | 0.33 | — | — | — |
| No. of samples | = 18 | 17 | 18 | 14 | 14 |
| Mean | = 1.43 | 0.97 | 0.60 | 0.44 | 0.35 |
| S.e. of the mean | = 0.29 | 0.23 | 0.10 | 0.16 | 0.07 |
| No. of paired samples | | 13 | 13 | 11 | 12 |

of AG and HC was $9.09 \pm 1.3\,\mu g\,ml^{-1}$, after 5 months of therapy. Allowing for the greater mean time interval between drug administration and collection of blood samples, our results for mean serum AG concentration, using a high pressure liquid chromatographic method, are very similar to those recorded by Murray *et al.* (1979).

Murray *et al.* (1979) also suggested that AG can induce its own metabolism and that induction is probably complete within one week of starting therapy. Our observations that serum AG concentrations increased in an approximate linear relationship with increasing dose suggest that if induction occurs then this is completed within the first month of therapy. However, NAG concentrations increased significantly more slowly, leading to a fall in the NAG:AG ratio during therapy, although the ratio appeared to stabilise after approximately six months of therapy. The fall in the NAG:AG ratio during chronic therapy suggests that alteration in the metabolism of NAG may occur during prolonged treatment. The cause of this alteration is not known, but it may be that the production of NAG is rate-limited. The metabolism of other compounds, that are acetylated, such as isoniazid, has been shown to be rate-limited (Thom *et al.*, 1981). Recently however, a novel metabolite of AG, hydroxylaminoglutethmide (hydroxyl-AG), has been described (Jarman *et al.*, 1983). This report suggested that hydroxyl-AG may be an induced metabolite as it is only formed during chronic therapy. Moreover, the data suggested that hydroxyl-AG may be formed at the expense of NAG. It is possible therefore, that the fall in the NAG:AG

**Table IV** Time intervals (minutes) between dose and blood sample in 24 patients receiving increasing doses of AG. (AG = aminoglutethimide; HC = hydrocortisone; b.d. = twice daily).

| | *Dose of AG (mg)* | | | | |
|---|---|---|---|---|---|
| *Patient No.* | *62.5 b.d.* | *125 b.d.* | *250 b.d.* | *500 b.d.* | *500 b.d. + HC* |
| | *Dose – sampling time intervals (min)* | | | | |
| 1 | 30 | 70 | — | 65 | — |
| 3 | 225 | 285 | 300 | — | — |
| 4 | 270 | — | — | — | 190 |
| 5 | 285 | 300 | 285 | — | — |
| 7 | — | — | 465 | 390 | 420 |
| 8 | 225 | 188 | 225 | 190 | 178 |
| 9 | 100 | 135 | 120 | 128 | 120 |
| 10 | 105 | — | — | 105 | 140 |
| 12 | 360 | — | 360 | — | — |
| 15 | — | 350 | 420 | 420 | 434 |
| 16 | — | 105 | 165 | 180 | — |
| 17 | 10 | 45 | — | — | 27 |
| 19 | 333 | 350 | 300 | 240 | 320 |
| 21 | 345 | 300 | 325 | 340 | 336 |
| 22 | 300 | 263 | 315 | — | — |
| 23 | 75 | 30 | 75 | 120 | 150 |
| 25 | 330 | — | 300 | 355 | 360 |
| 26 | 120 | 160 | 165 | — | — |
| 29 | — | 360 | 390 | — | — |
| 30 | 90 | 90 | 30 | — | — |
| 31 | — | — | 360 | 360 | 340 |
| 32 | — | 225 | — | 170 | 158 |
| 33 | 315 | — | 225 | 315 | 315 |
| 34 | 420 | 380 | — | — | — |
| No. of samples | = 18 | 17 | 18 | 14 | 14 |
| Mean | = 219 | 214 | 268 | 241 | 249 |
| S.e. of the mean | = 128 | 120 | 119 | 119 | 126 |

ratio might reflect increasing production of hydroxyl-AG.

Further studies, however, will be necessary to confirm that the NAG:AG ratio falls during chronic therapy and to establish the mechanism and possible clinical significance of this alteration.

RS-H was in receipt of a Research Fellowship awarded by the Clinical Research Committee of the Royal Marsden Hospital during this study. We are grateful to Miss S. Beehnick and Mrs L. Carr for help in sample and data collection and Miss J. Hood for preparation of the manuscript.

**Table V** NAG:AG ratios in 15 patients receiving AG for more than 4 months. Numbers in parentheses represent the dose-sampling intervals in minutes (NAG = N-acetylaminoglutethimide; AG = aminoglutethimide).

| *Patient No.* | *Months of therapy* 4 | 5 | 6 | 7 | ≥8 |
|---|---|---|---|---|---|
| | *NAG:AG Ratio* | | | | |
| 7 | 0.18(390) | 0.26(420) | — | 0.21(420) | — |
| 8 | 0.40(190) | 0.42(200) | 0.58(165) | — | 0.52(170) |
| 9 | 0.89(135) | 0.26(120) | 0.16(135) | 0.15(120) | 0.20(120) |
| 10 | 0.58(105) | 0.66(140) | — | — | — |
| 15 | 0.15(420) | 0.18(420) | 0.12(420) | 0.12(460) | 0.12(435) |
| 16 | 0.36(105) | — | 0.27(165) | 0.17(180) | 0.23(105) |
| 17 | 0.23 (30) | — | 0.15 (30) | 0.15 (30) | 0.13 (20) |
| 19 | 2.42(240) | 0.52(255) | 0.40(295) | 0.46(300) | 0.37(330) |
| 21 | 0.23(325) | 0.15(340) | 0.11(290) | 0.09(320) | 0.13(420) |
| 23 | 0.15(150) | — | — | — | 0.12(320) |
| 25 | 0.37(355) | 0.41(360) | 0.38(360) | — | — |
| 30 | 1.82(120) | 1.54(105) | — | — | 1.57(120) |
| 31 | 0.33(360) | 0.28(360) | 0.40(330) | — | 0.29(330) |
| 32 | — | 0.20(170) | 0.20(165) | 0.21(195) | 0.18(130) |
| 33 | 0.71(315) | 1.31(300) | 0.84(330) | — | — |
| No. of samples | = 14 | 12 | 11 | 8 | 11 |
| Mean | = 0.63(231) | 0.52(266) | 0.33(244) | 0.20(253) | 0.35(227) |
| S.e. of the mean | = 0.18 (34) | 0.13 (34) | 0.07 (36) | 0.04 (52) | 0.13 (43) |

### References

ADAM, A.M., BRADBROOK, I.D. & ROGERS, H.J. (1985). High performance liquid chromatographic assay for simultaneous estimation of aminoglutethimide and acetylaminoglutethimide in biological fluids. *Cancer Chemother. Pharmacol.*, (in press).

BRODIE, A.M.H. (1982). Overview of recent development of aromatase inhibitors. *Cancer Res.*, **42**, (suppl.), 3312s.

CASH, R., BROUGH, A.J., COHEN, M.N.P. & SOTOL, P.S. (1967). Aminoglutethimide (Elipten-Ciba) as an inhibition of adrenal steroidogenesis: Mechanism of action and therapeutics trial. *J. Clin. Endocrinol. Metab.*, **27**, 1239.

CASH, R., BROUGH, A.J. COHEN, M.N.P. & SOTAL, P.S. (1967). Aminoglutethimide (Eliptn-Cila) as an in onhibition of adrenal steroidogenesis: Mechanism of action and therepeutics trial. *J. Clin. Endocrinol. Metal.*, **27**, 1239.

COOMBES, R.C., JARMAN, M., HARLAND, S. & 7 others (1980). Aminoglutethimide: Metabolism and effects on steroid synthesis *in vivo*. *J. Endocrinol.*, **87**, 31.

CUPPLES, L.A., HEEREN, T., SCHATZKIN, A. & COLTON, T. (1984). Multiple testing of hypotheses in comparing two groups. *Ann. Int. Med.*, **100**, 122.

DIXON, W.J. (ED.) (1981). *BMDP Statistical Software*, p. 235, University of California Press, Berkeley.

DOUGLAS, J.S. & NICHOLLS, P.J. (1965). The urinary excretion of aminoglutethimide in man. *J. Pharm. Pharmacol.*, **17**, (suppl.), 115s.

DOUGLAS, J.S. & NICHOLLS, P.J. (1972). The partial fate of aminoglutethimide in man. *J. Pharm. Pharmacol.*, **24**, (Suppl.), 150p.

GRAVES, P.E. & SALHANICK, H.A. (1979). Stereoselective inhibition of aromatase by enantiomers of aminoglutethimide. *Endocrinology*, **105**, 52.

GRODIN, J.M., SIITERI, P.K. & MACDONALD, P.C. (1973). Source of estrogen production in postmenopausal women. *J. Clin. Endocrinol. Metab.*, **36**, 207.

HARRIS, A.L., POWLES, T.J. & SMITH, I.E. (1982). Aminoglutethimide in the treatment of advanced postmenopausal breast cancer. *Cancer Res.*, **42**, (suppl.), 3405s.

HARRIS, A.L., DOWSETT, M., SMITH, I.E. & JEFFCOATE, S.L. (1983). Endocrine effects of low dose aminoglutethimide in advanced postmenopausal breast cancer. *Br. J. Cancer*, **47**, 621.

JACKSON, L., HOMEIDA, M. & ROBERTS, C.J.C. (1978). The features of hepatic enzyme induction with glutethimidine in man. *Br. J. Clin. Pharmacol.*, **6**, 525.

JARMAN, M., FOSTER, A.B., GOSS, P.E., GRIGGS, J. HOWE, I. & COOMBES, R.C. (1983). Metabolism of aminoglutethimide in humans: Identification of hydroxylaminoglutethimide as an induced metabolite. *Biomed. Mass. Spec.*, **10**, 620.

KAHNT, F.W. & NEHER, R. (1966). On the adrenal biosynthesis of steroids *in vitro*. III. Selective inhibition of adrenocortical function. *Helv. Chem. Acta.*, **49**, 725.

MacDONALD, P.C., ROMBAUT, R.P. & SIITERI, P.K. (1967). Plasma precursors of estrogen. I. Extent of conversion of plasma Δ4 androstenedione to estrone in normal males and nonpregnant, normal, castrate and adrenalectomized females. *J. Clin. Endocrinol. Metab.*, **27**, 1103.

MURRAY, F.T., SANTNER, S., SAMOJLIK, E.A. & SANTEN, R.J. (1979). Serum aminoglutethimide levels: Studies of serum half-life, clearance and patient compliance. *J. Clin. Pharmacol.*, **19**, 704.

SANTEN, R.J., SANTNER, S., DAVIS, B., VELDHUIS, J., SAMOJLIK, E. & RUBY, E. (1978). Aminoglutethimide inhibits extraglandular estrogen production in postmenopausal women with breast carcinoma. *J. Clin. Endocrinol. Metab.*, **47**, 1257.

SANTEN, R.J. & MISBIN, R.I. (1981). Aminoglutethimide: Review of pharmacology and clinical use. *Pharmacol. Ther.*, **1**, 95.

SANTEN, R.J., WORGUL, T.J., LIPTON, A. & 4 others (1982). Aminoglutethimide as treatment of post-menopausal women with advanced breast carcinoma. *Ann. Int. Med.*, **96**, 94.

SMITH, I.E., FITZHARRIS, B.M., McKINNA, J.A. & 6 others (1978). Aminoglutethimide in the treatment of metastatic breast carcinoma. *Lancet*, **ii**, 646.

STUART-HARRIS, R., DOWSETT, M., D'SOUZA, A & 6 others (1984). Low dose Aminoglutethimide in treatment of advanced breast cancer. *Lancet*, **ii**, 604.

STUART-HARRIS, R., DOWSETT, M., D'SOUZA, A. & 4 others (1985). Endocrine effects of low dose aminoglutethimide as an aromatase inhibitor in the treatment of breast cancer. *Clin. Endocrinol.*, (in press).

THOM, S., FARROW, P.R., SANTOSO, B., ALBERTI, K.G.M.M. & RAWLINS, M.D. (1981). Effects of oral glucose in lime juice on isoniazid kinetics. *Br. J. Clin. Pharmacol.*, **11**, 423p.

THOMPSON, T.A., VERMEULEN, J.D., WAGNER, W.E. & LE SHER, A.R. (1981). Aminoglutethimide bioavailability, pharmacokinetics and binding to blood constituents. *J. Pharm. Sci.*, **70**, 1040.

VERMEULEN, A., PARIDAENS, R. & HEUSON, J.C. (1983). Effects of aminoglutethimide on adrenal steroid secretion. *Clin. Endocrinol.*, **19**, 673.